# Standing-Wave Feeding for High-Gain Linear Dielectric Resonator Antenna (DRA) Array

**DOI:** 10.3390/s22083089

**Published:** 2022-04-18

**Authors:** Kerlos Atia Abdalmalak, Ayman Abdulhadi Althuwayb, Choon Sae Lee, Gabriel Santamaría Botello, Enderson Falcón-Gómez, Luis Emilio García-Castillo, Luis Enrique García-Muñoz

**Affiliations:** 1 Department of Signal Theory and Communications, Carlos III University of Madrid, 28903 Madrid, Spain; efalcon@pa.uc3m.es (E.F.-G.); luise@tsc.uc3m.es (L.E.G.-C.); legarcia@ing.uc3m.es (L.E.G.-M.); 2 Electrical Engineering Department, Aswan University, Aswan 81542, Egypt; 3 Department of Electrical Engineering, College of Engineering, Jouf University, Sakaka 72388, Saudi Arabia; aaalthuwayb@ju.edu.sa; 4 Electrical and Computer Engineering Department, Southern Methodist University, Dallas, TX 75205, USA; csl@lyle.smu.edu; 5 Electrical, Computer and Energy Engineering Department, University of Colorado, Boulder, CO 80309, USA; gasantam@pa.uc3m.es

**Keywords:** antenna array feeds, dielectric resonator antenna (DRA), linear antenna arrays, standing wave, high-gain antennas, high radiation efficiency, 3D printing

## Abstract

A novel feeding method for linear DRA arrays is presented, illuminating the use of the power divider, transitions, and launchers, and keeping uniform excitation to array elements. This results in a high-gain DRA array with low losses with a design that is simple, compact and inexpensive. The proposed feeding method is based on exciting standing waves using discrete metallic patches in a simple design procedure. Two arrays with two and four DRA elements are presented as a proof of concept, which provide high gains of 12 and 15dBi, respectively, which are close to the theoretical limit based on array theory. The radiation efficiency for both arrays is about 93%, which is equal to the array element efficiency, confirming that the feeding method does not add losses as in the case of standard methods. To facilitate the fabrication process, the entire array structure is 3D-printed, which significantly decreases the complexity of fabrication and alignment. Compared to state-of-the-art feeding techniques, the proposed method provides higher gain and higher efficiency with a smaller electrical size.

## 1. Introduction

For decades, dielectric resonators (DRs) have been widely utilized as tuners or amplifiers in microwave-circuit applications [1] due to their high Q-factors. The utilization of antennas had to wait for a long time, until Long et al. presented the first cylindrical dielectric resonator antenna (DRA) in 1983 [2]. Ever since then, dielectric antennas have been intensively studied as potential substitutions of traditional less efficient metal radiators, which have serious problems at high frequencies [3,4,5]. There are some metal antennas such as lens [6] and slot [7] antennas that can avoid such losses and provide high gain with high efficiency, but they still have the drawbacks of being bulky and complex, respectively. From this perspective, DRAs come with numerous beneficial features such as high radiation efficiency, easy excitation scheme, light weight, and small size [8,9,10,11]. One of the main limitations of the DRA is its relatively low gain [12]; hence, dielectric resonator antenna (DRA) arrays are a preferred choice for many applications [13,14].

Several excitation schemes are used to feed DRA arrays [15], such as series or corporate microstrip lines [16,17], standard rectangular waveguide (RWG) [18,19,20,21], substrate integrated waveguide (SIW) [22,23,24], and dielectric image waveguide (DIG) [25,26,27]. Traditional corporate-feeding networks have a number of power dividers that cause spurious radiation, resulting in high losses [28]. Because of this, new feeding techniques focus on avoiding such losses, for example, in [29], the authors proposed the use of a single microstrip line to feed the whole array. Although this feeding technique is simple and of low cost, the resulting gain is relatively small (15dBi using 9 elements), and its efficiency is below 80% [30]. Efficiency can be enhanced in the case of feeding only a single element and turning the rest of the array elements into parasitic elements, as in [31]; however, field distribution is not the same for all elements, which results in relatively low gain. For example, in [32], a gain of about 6.6dBi was obtained using five elements. The RWG feeding scheme has two major drawbacks, high production costs and a bulky structure, which hinder array design [33]. Another feeding method is SIW, which results in significant leakage losses through multiple metallic vias [22] and extra complexity in the design. Lastly, in the DIG feeding scheme, considerable backradiation is a major issue [25]. Additionally, tapered rectangular waveguides are needed to launch the DIG that, in turn, renders the structure bulky and increases the fabrication cost [34].

Although state-of-the-art feeding methods provide some good characteristics, there is still a main research gap to find a novel feeding method that simultaneously illuminates the use of power dividers, transitions, or launchers with keeping uniform excitation to the array elements and low losses in the feeding network. This results in a high-gain DRA array with high efficiency, and renders the design simple, compact, and inexpensive, which are essential parameters for wireless applications such as sensing applications and 5G base station antennas. Hence, this paper presents a novel feeding scheme for linear arrays based on the standing-wave concept [35], compared to our previous standing-wave DRA array presented in [36] where the standing wave was formed by dielectric bridges with metal cover between elements. Here, it is formed within discrete metallic patches printed in the same substrate layer. This ensures more uniform excitation between elements, and could thus achieve performance close enough to the theoretical limit of the array, and achieve a similar gain with a lower number of elements compared to [36], which, in turn, would significantly decrease the size of the solution, as is demonstrated in Section 5.

This study is organized as follows. The detailed design procedure of the array element which acts as the unit cell of the array is presented in Section 2. A two-element array design based on a standing-wave feed is explained in Section 3 with the electric field distribution in the feeding network to confirm the forming of standing waves. Then, the concept is extended to a larger array of four elements in Section 4 with the theoretical calculations of the array factor to validate the feeding concept and confirm the uniform excitation of the array elements using the novel feeding method. Fabrication and measured results are given in Section 5 along with a comparison to DRA arrays fed by state-of-the-art feeding methods from different points of view. Lastly, the main remarks and future work are concluded in Section 6.

## 2. Design of DRA Array Unit Cell

**Figure 1:**
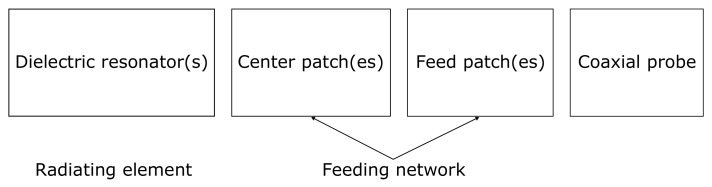
Block diagram of DRA array based on the proposed feeding method.

Figure 1 presents a general high-level block diagram of DRA array fed by the proposed standing wave feeding method. The design consists of discrete metallic patches called feed and center patches transferring the signal from the coaxial probe to the dielectric resonator which acts as the radiating element. The feed patch was designed to form a standing wave under the stripline to feed the antenna element. Then, another smaller patch (called a center patch) is inserted with a gap distance to control the field excitation under the dielectric resonator. Lastly, a dielectric resonator (radiating element) is introduced above the center patch. The total distance between neighboring elements is about one and a half guided wavelengths for maximal array gain. It is clear here that the proposed feeding method excited the radiating element without the need for any power dividers, transitions, or launchers, which would significantly affect DRA array performance, as is demonstrated in the rest of the paper.

**Figure 2:**
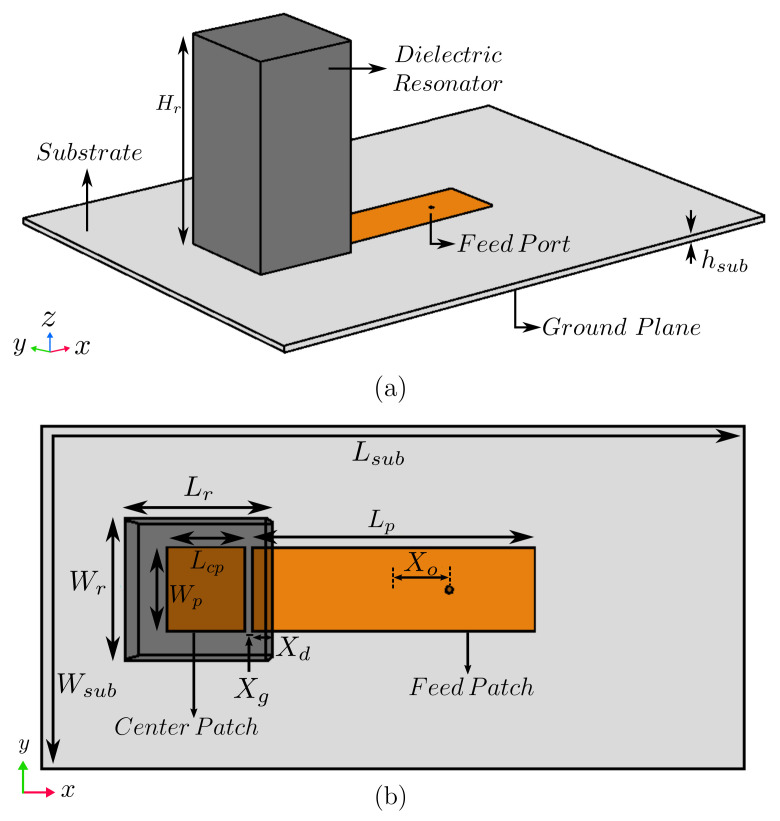
(**a**) 3D view of DRA Unit cell and (**b**) top view with transparent resonator.

As an initial step of the array design, the radiating element was designed, which is a rectangular dielectric resonator as shown in Figure 2. Here, a higher-order mode ${\mathrm{TE}}_{113}$ is excited at a frequency of 3.9GHz to obtain higher gain than that of the fundamental mode of ${\mathrm{TE}}_{111}$ (enhancement of about 2dB). The optimized dimensions of the resonator were $L_{r}=26.2\;\mathrm{mm}$, $W_{r}=26.2\;\mathrm{mm}$, and $H_{r}=45.8\;\mathrm{mm}$. It was composed of polylactic acid (PLA) with dielectric constant $\varepsilon_{r}=3.549$ [37,38]. High-permittivity resonators ($\varepsilon_{r}$ up to 140 [12]) can be used if compact sizes are needed, but at a high cost. The feed-network substrate was Arlon 25N with dielectric constant $\varepsilon_{r}\left(\mathrm{sub}\right)=3.38$, loss tangent $\delta_{\mathrm{sub}}=0.0015$, thickness $h_{\mathrm{sub}}=1.5\;\mathrm{mm}$, and surface area $120\times 160\;{\mathrm{mm}}^{2}$ ($W_{\mathrm{sub}}\times L_{\mathrm{sub}})$.

The next step is to add the feeding network to have the unit cell for the linear DRA array. To create such a unit cell, feed and center patches with dimensions of 61.5×17mm ($L_{p}\times W_{p}$) and 15×17mm ($L_{\mathrm{cp}}\times W_{p}$) were introduced with a gap of $X_{g}=1.4\;\mathrm{mm}$ between them, as shown in Figure 2. The designed dielectric resonator was placed above the center patch with an overlapping distance ($X_{d}=4.2\;\mathrm{mm}$) between resonator and feed patch, which is needed for sufficient coupling between resonator and feed patch. A 50Ω coaxial probe was placed at an offset distance of $X_{o}=7.6\;\mathrm{mm}$ from the center of the feed patch for proper impedance matching.

**Figure 3:**
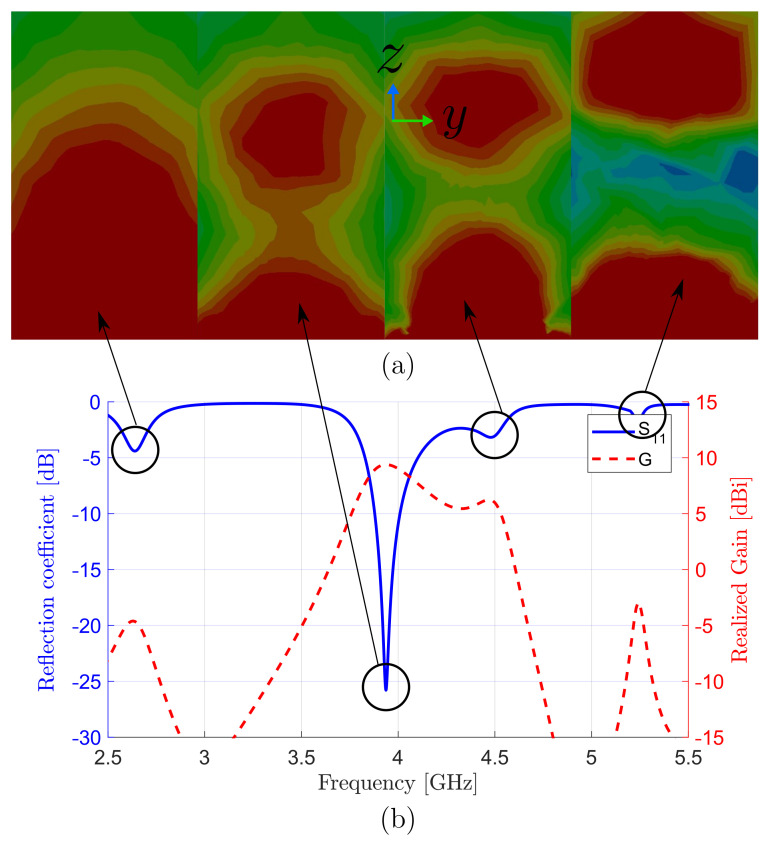
(**a**) Field distribution of ${\mathrm{TE}}_{113}$ mode at 3.9GHz and neighboring resonant modes inside DRA. (**b**) Simulated $S_{11}$ and gain vs. frequency of antenna element.

The simulated field distribution of ${\mathrm{TE}}_{113}$ mode in the H-plane (y–z plane) inside the DRA at the resonance frequency of 3.9GHz is shown in Figure 3, along with the reflection coefficient and gain as a function of frequency. Figure 3a shows interested mode ${\mathrm{TE}}_{113}$ excited at 3.9GHz and three other neighbor resonant modes at frequencies of 2.6, 4.5, and 5.2GHz, which correspond to ${\mathrm{TE}}_{111}$, ${\mathrm{TE}}_{113}$, and a hybrid mode, respectively. The design and coaxial probe can be easily readjusted to match any of these resonances. The second and third modes were a single ${\mathrm{TE}}_{113}$, which was split into two resonances due to the impedance mismatching. Hence, by changing the antenna element, matching can be enhanced, and modes come closer which needs a larger bandwidth; however, this is omitted here, as the main contribution for the paper is array feeding and not element design. The antenna produced a peaked gain of 9.3dBi in the boresight direction ($\theta =0^{\circ }$), which outperformed DRA elements operating at ${\mathrm{TE}}_{113}$ [39,40]. This illustrates the efficient feeding scheme of the standing wave, even for a single-element DRA antenna.

The dimensions were optimized for maximal gain using high-frequency structure simulator (HFSS) [41]. The design was simulated under the driven modal on the basis of the finite element method (FEM) and adaptive meshing. The convergence condition was set to achieve target delta S-parameters below 2%, which implied dividing the structure into 20 to 40 thousand tetrahedral meshes for single and four elements, respectively. The used boundary conditions are absorbing boundary condition (ABC) for a surrounding air box that is a quarter wavelength farther away than the antenna edges. Metal feed and center patches were approximated with the perfect electric conductor (PEC) boundary condition during optimization, and were replaced by normal copper units with a thickness of 35 μm at the final simulations.

## 3. Two-Element Standing-Wave Linear Array

In this section, a 1×2 linear array based on the standing-wave feed method is presented. To form the two-element array, the unit cell in Figure 2 is replicated twice. Since the second cell was the end element of the array, there was no need to add another feed patch at the right of the second element, as the feed patch at the middle would already feed the two elements. Hence, for a general 1×N element array, we needed *N* center patches lying underneath each dielectric resonator and N−1 feed patches to uniformly deliver energy to all elements.

**Figure 4:**
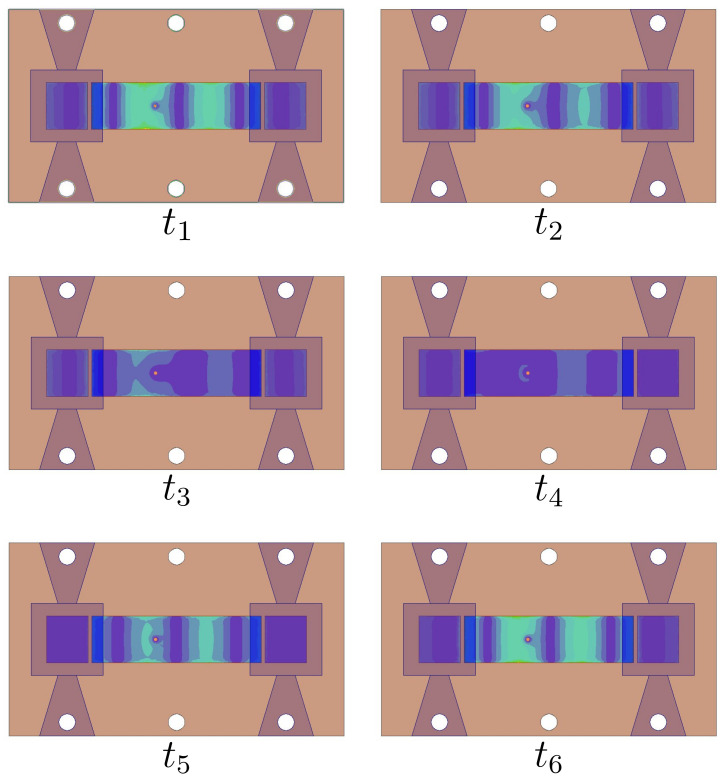
Snapshots of electric field distribution in proposed array demonstrating standing-wave excitation in the feed network.

**Figure 5:**
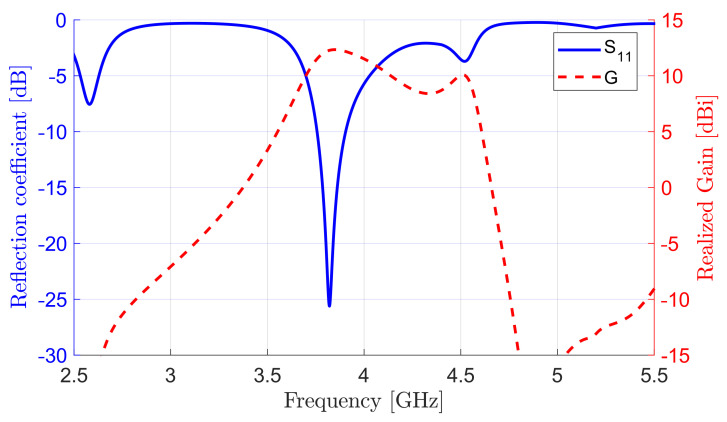
Simulated $S_{11}$ and gain vs. frequency of the proposed two-element standing-wave DRA array.

A standing wave is formed when two waves travel in opposite directions with an equal magnitude within the feeding network, where null locations do not move. Figure 4 shows the fixed places of null field points in the feeding network. Simulation results are shown in Figure 5, where a maximal realized gain of about 12dBi was obtained at the resonant frequency. Such a high gain is 2.7dB over the single-element gain.

## 4. Four-Element Standing-Wave Linear Array

**Figure 6:**
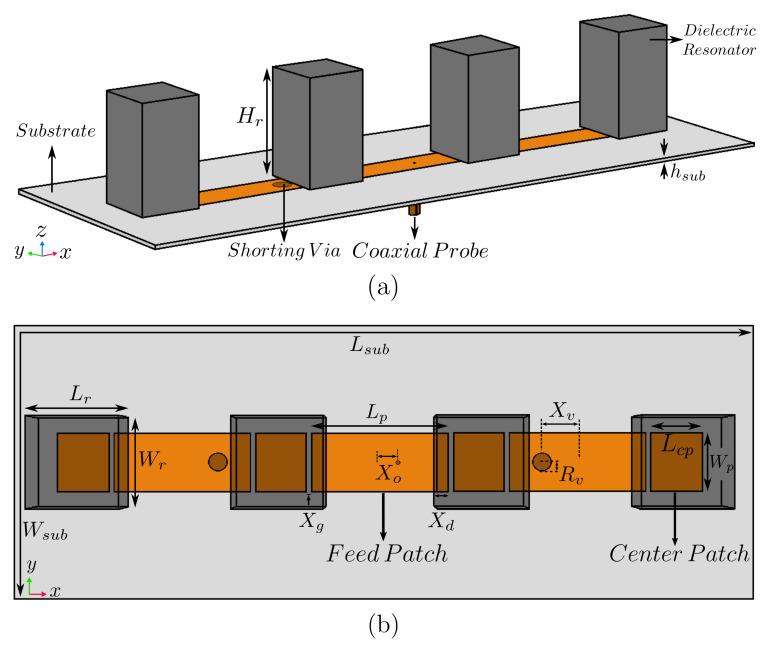
Schematic diagram of proposed four-element standing-wave DRA array: (**a**) 3D view and (**b**) top view with transparent resonators.

The proposed configuration was extended to create a four-element linear array by adding two more unit cells to the two-element design as shown in Figure 6. Considering that the number of elements (*N*) was 4, and following our discussion above, the four-element array consists of 4 dielectric resonators lying above 4 center patches with 3 connecting feed patches. The optimized geometrical parameters, as summarized in Table 1, remained almost the same as the number of array elements increased. Such a feature provides a convenient design procedure for various array sizes.

**Figure 7:**
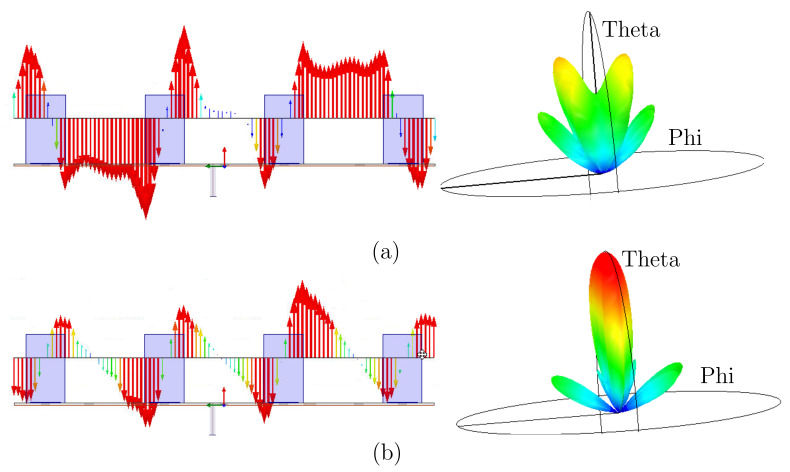
Field distribution and radiation pattern of proposed four-element DRA array (**a**) without and (**b**) with shorting pins.

In a standing-wave array, the entire array acts as a single large resonator; hence, more than one mode can be strongly excited near the operating frequency, especially in a large array. Unfortunately, some of the excited modes do not produce radiation in the intended direction. Figure 7a presents the field distribution and 3D radiation pattern of the array, being red, yellow, green, and blue colors represent the strength from the max to the minimum. It shows such an example where the fields of the two end elements are in the opposite direction to those of the two inner elements, producing diminished radiation in the boresight direction. To eliminate such unwanted modes, shorting pins are used. Figure 7b shows that the added pins suppress those undesirable modes to give more uniform fields among all array elements for maximal directivity.

**Figure 8:**
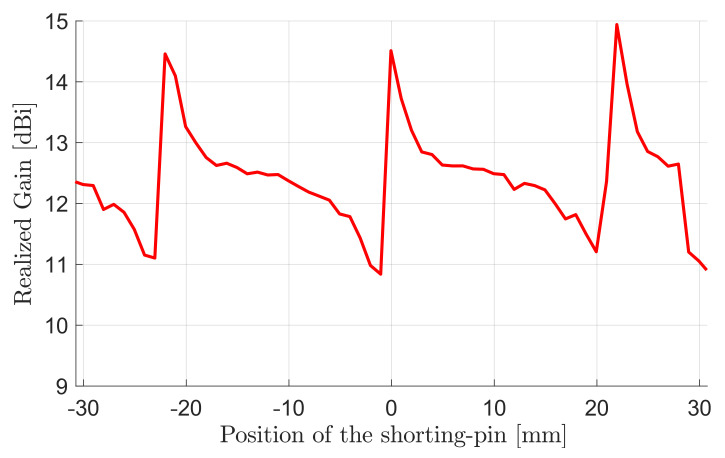
Gain of four-element antenna vs. position of shorting pin along feed patch.

Figure 8 presents the effect of the shorting-pin position on the array gain. The three pin positions for maximal gain indicate three null electric-field points of the desired mode under the patch because the presence of the pins does not affect the field distribution optimal for gain, but puts down other undesirable modes with finite field strength at the pin location. The radius of the shorting pins was also optimized, so that the undesired modes become eliminated as much as possible, while the selected mode is least affected by the vias.

**Figure 9:**
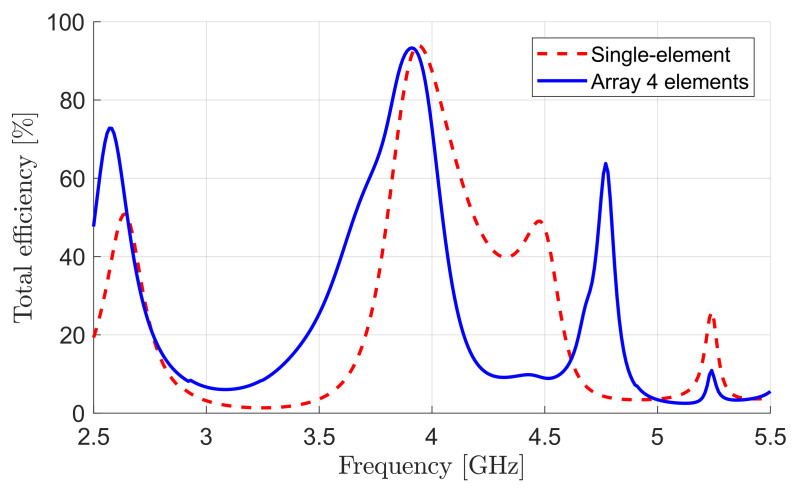
Efficiency of proposed DRA array.

The simulated total efficiency of the four-element design is shown in Figure 9. High efficiency of about 93% was achieved for the four-element array at the resonant frequency, which was only 1% less than that of the single-element design. This demonstrates the benefit of the proposed feeding technique compared to other feeding methods that introduce extra losses in the array structure, resulting in a reduction in antenna efficiency.

**Figure 10:**
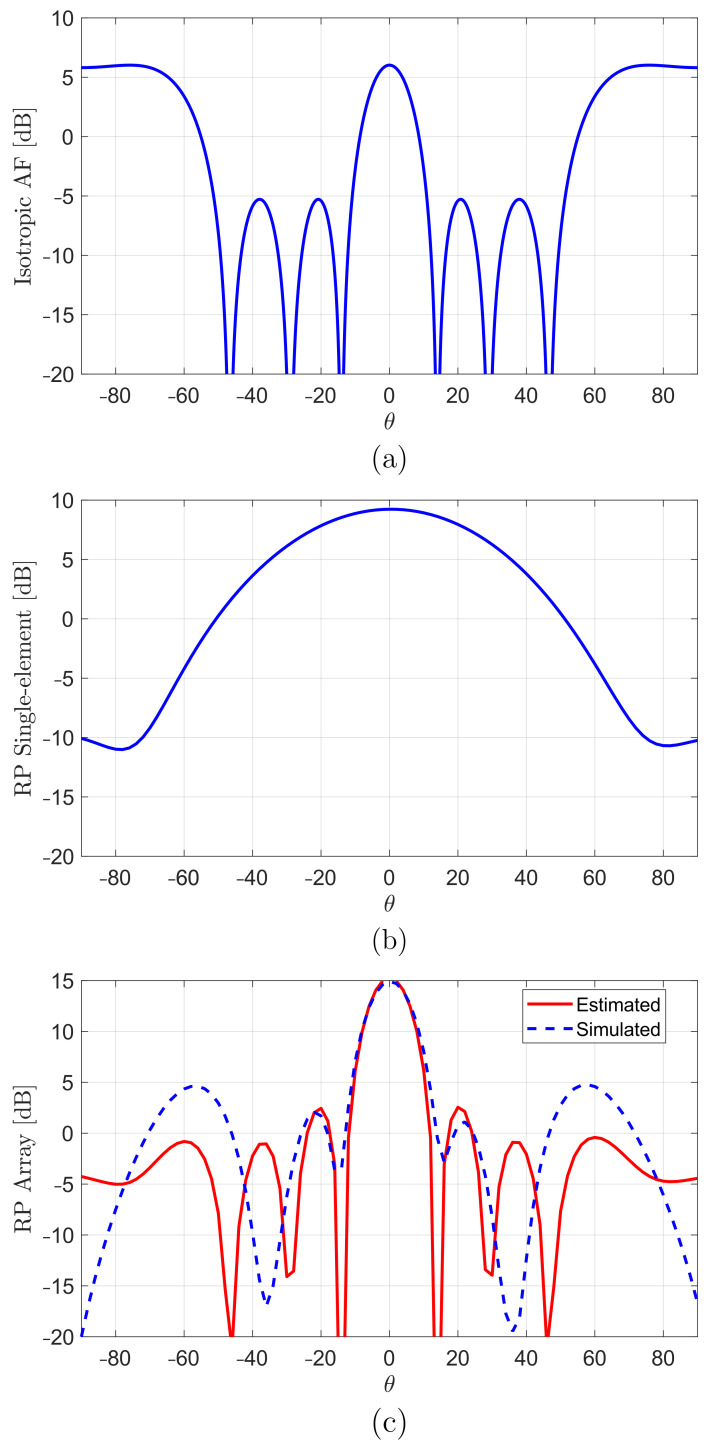
(**a**) Theoretical array factor of four isotropic sources; (**b**) simulated element factor; and (**c**) simulated and estimated radiation pattern of proposed DRA array.

For comparison purposes and to validate the performance of the proposed array feeding method, the array factor for an ideal array was estimated, as is shown in Figure 10a. Hence, four equispaced isotropic elements with the same element spacing as the proposed array and fed with the same amplitude and phase. With this, an upper limit of the gain improvement could be estimated.

Such array factor can be added (in dB) to the actual radiation patterns of the array element (Figure 10b) to compute the estimated total pattern of the array in such an ideal case (uniform feeding to all antenna elements). Figure 10c demonstrates that the proposed feeding method efficiently excites the four elements as the actual simulated radiation patterns of the array are in excellent agreement with the theoretical estimated especially near the boresight. It is clear that the simulated one provided a peak gain of about 15dBi, which was almost equal to the upper theoretical limit. in other words, the gain enhancement for the four-element array over that of a single element is 5.7dB, which is approximately equal to the theoretical array gain of a four isotropic-element array with the same element spacing of 1.03 $\lambda_{0}$ [42].

## 5. Fabrication and Measurements

**Figure 11:**
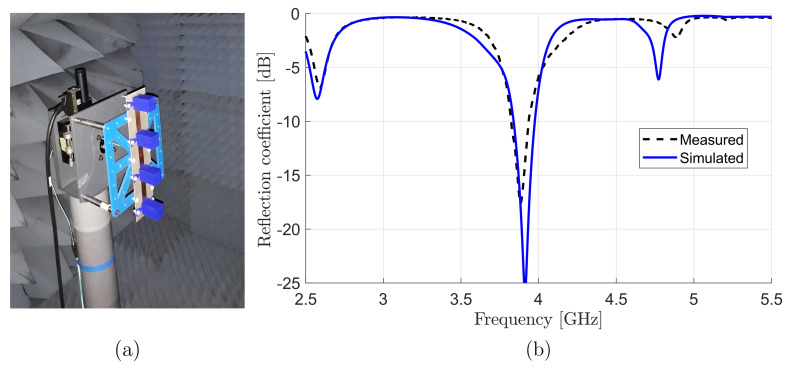
(**a**) Manufactured prototype of four-element DRA array. (**b**) Measured and simulated $S_{11}$ of manufactured four-element standing-wave DRA array prototype.

A four-element standing-wave DRA array was fabricated (Figure 11a) using 3D printing, which is a promising technique for antennas at microwave ranges [43,44]. Two small arms were included in each dielectric element to ensure accurate alignment. Simulated and measured reflection coefficients, and realized gain were in excellent agreement, as shown in Figure 11b.

**Figure 12:**
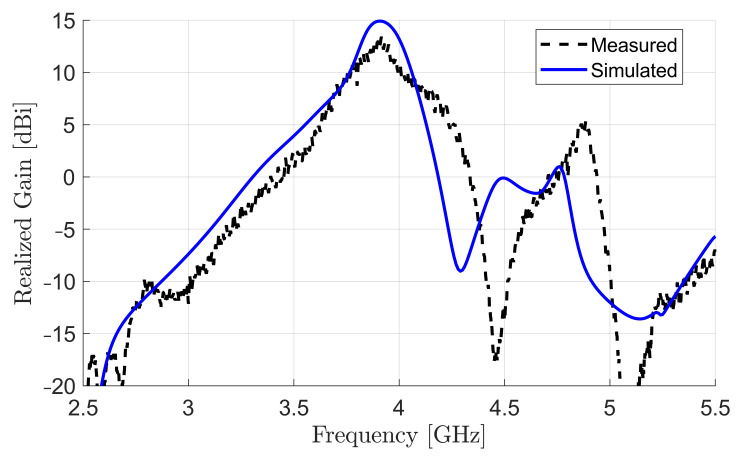
Simulated and measured realized gain of four-element standing-wave DRA array.

For gain and radiation pattern measurements, the DRA array was adjusted in an anechoic chamber in the receiving mode with transmitting the signal from the vector network analyzer (VNA) through a standard commercial horn. The spacing between the two antennas was selected to ensure that the tested antenna would be in the far-field region. The signal was amplified using a power amplifier before delivering to the horn to compensate the free space and cables losses, and ensure good receiving power levels above the noise floor of the measurement setup [45]. On the basis of the direct comparison method using a third horn with known gain vs. frequency values, the realized gain as a function of frequency of the proposed DRA array was measured, as shown in Figure 12. Compared to a maximal simulated realized gain of 15dBi, the measured gain showed a value of 14dBi at the resonant frequency. The difference in gains was due to uncertainties in material properties, as is explained later in this section. The array had an impedance and 3 dB gain bandwidth product of about 4%, which was equal to those of the single-element antenna. In other words, the proposed array feeding method did not reduce either impedance or gain bandwidths.

**Figure 13:**
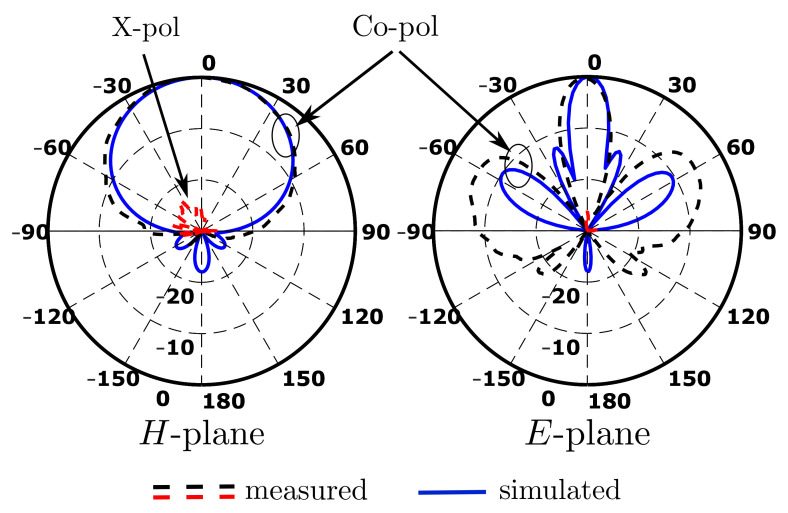
Simulated and measured radiation patterns of four-element standing-wave DRA array.

The simulated and measured radiation patterns of the dielectric array at the resonance frequency in both E- and H-planes were in relatively good agreement (Figure 13). The simulated sidelobe level was about −10dB, which was the best achievable value of DRA working in ${\mathrm{TE}}_{113}$ mode [40,46]; however, the measured sidelobe level was lower than that of the simulated. Investigating the possible sources for the mismatching more, and Figure 11 and Figure 12 show that there was a frequency shift up to higher frequency. From this point, it appeared that the reason behind this mismatching was the difference in the dielectric properties of polylactic acid (PLA) due to 3D printing conditions.

Despite the fact that PLA dielectric constant should be about 3.5 at a frequency range around 4GHz [37,38], the value practically depends on 3D manufacturing properties such as printing temperature [47] and printing resolution [48]. Hence, by considering actual manufacturing conditions (printing temperature of 220 °C and layer thickness of 50 μm) and at the used frequency band, the dielectric constant would be affected to be slightly smaller (around 2.7) [47,48,49,50].

**Figure 14:**
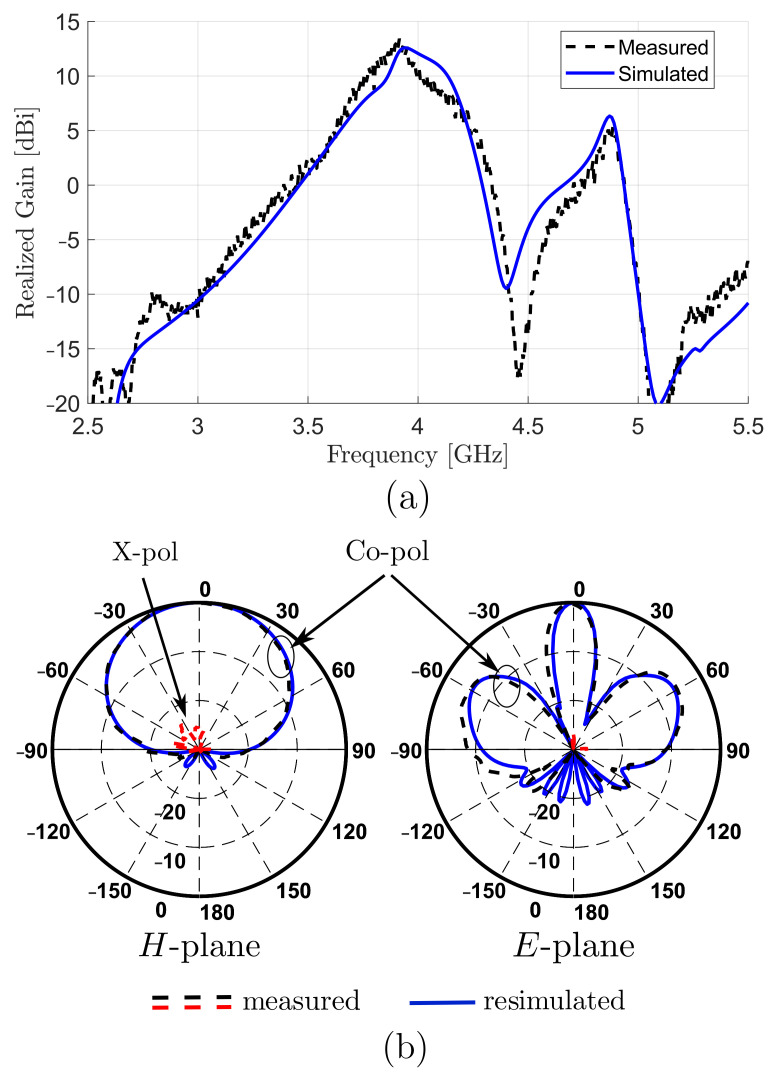
Resimulated and measured: (**a**) realized gain; (**b**) radiation patterns after considering mismatching in dielectric characteristics.

To see the effect of such changes in the dielectric constant, the array was resimulated using the new estimated dielectric constant. The resimulated and measured gain and radiation patterns were in excellent agreement, as shown in Figure 14.

Table 2 shows a comparison of the four-element dielectric array based on the proposed feeding scheme with those of the state-of-the-art feeding techniques for DRA arrays with highlighting their drawbacks in red.The proposed feeding method showed a significant gain improvement of about 3 to 6.5dB compared with recently reported designs of other feeding schemes. It also remarkably enhanced efficiency compared to standard microstrip lines or DIG feeds. Moreover, in comparison to DIG or SIW, the proposed feeding produced a comparable gain that was provided by the 4 times greater number of elements, which resulted in a very compact design of about 10% of the size of other feeds, in addition to a decrease in cost and complexity. Even for our previous work based on standing-wave feeding [36] which successfully achieved most of the previously mentioned advantages, the feed in this paper is more compact as a similarly high gain of 14dBi was achieved here by 4 elements (2$\lambda^{3}$), while 9 elements ($18.7\lambda^{3}$) are needed for the previous feeding method to achieve a comparable gain of 14dBi. Regarding the impedance and 3 dB gain bandwidth (BW) product, the proposed feed showed comparable BW to SIW, microstrip lines, and standing-wave feeding methods with lower BW compared to DIG and parasitic methods. However, due to the previously mentioned advantage, the proposed feeding still presented the best overall performance. Additionally, as confirmed in Section 5, this feeding did not degrade array performance, so larger BW could easily be achieved by updating the array element using any standard DRA bandwidth enhancement techniques, as is briefly discussed in Section 6; the proposed array would then have as large a bandwidth as that of the array element.

## 6. Conclusions and Future Work

In this paper, a high-gain linear DRA array with a standing-wave feed network is presented without the need of using power dividers, transitions, or launchers. The proposed feed technique provided relatively high gain and excellent efficiency compared with those of standard feeding methods. Such attractive features come in addition to a simple and compact design with a straightforward impedance-matching procedure, similar to that of regular microstrip antennas. Arrays based on the proposed feeding method with a different number of elements were presented with manufacturing a four-elements array with low-cost 3D-printing technology. Good agreement was realized between the simulated and measured results with a peak measured gain of 14dBi. The small deviation between theory and experiments was investigated and was due to changes in the dielectric properties of the resonator material depending on 3D manufacturing properties.

Possible extensions for the work are divided into three main research lines. First, improving the bandwidth, which can be achieved by designing an array on the basis of the same feeding method but using different DRA array element shapes. For example, using an element consisting of multiple dielectric slabs instead of the standard rectangle one to increase the bandwidth (can provide an improvement around 3 times) or using metallic patches as the superstrate. Second, improving the gain even further by using pyramidal horns instead of rectangle resonators. Lastly, scaling the design to work at higher frequencies such as mm wave ranges.

## Figures and Tables

**Table 1:** Geometrical parameters of DRA array (unit: mm).

$L_{\mathrm{sub}}$	$W_{\mathrm{sub}}$	$h_{\mathrm{sub}}$	$L_{r}$	$W_{r}$	$H_{r}$	$L_{p}$
280	70	1.52	26.2	26.2	45.8	61.5
$L_{\mathrm{cp}}$	$W_{p}$	$X_{g}$	$X_{o}$	$X_{d}$	$R_{v}$	$X_{v}$
15	17	1.4	7.6	4.2	4	22

**Table 2:** Comparison of proposed standing-wave DRA array with arrays based on state-of-the-art feeding techniques.

Ref	DRA Array Feeding Method	No. of Elements	Gain @$f_{0}$ (dBi)	Antenna Size ($\lambda^{3}$)	Impedance/Gain BW Product (%)	Efficiency @$f_{0}$ (%)
[31]	Parasitic	3	9.25	0.89 (1.5×1×0.59)	9	92
[23]	SIW	4	10.6	1 (1.13×3.5×0.26)	5	93
[16]	Microstrip lines	4	10	0.9 (2.5×2.13×0.17)	3	85
[25]	DIG	7	7.61	15.4 (4.66×9.77×0.34)	10	64.6
[24]	SIW	8	11.5	11 (8.4×6.3×0.21)	2	85
[36]	Standing-wave	9	15	18.7 (4.11×3.73×1.22)	7	91
[25]	DIG	15	12.46	25.2 (4.66×16×0.34)	15	63
This work	Standing-wave	2	12	0.9 (0.91×1.58×0.61)	4	93
This work	Standing-wave	4	14	2 (0.91×3.64×0.61)	4	93

## Data Availability

Not applicable.
